# Folic acid reduces doxorubicin‐induced cardiomyopathy by modulating endothelial nitric oxide synthase

**DOI:** 10.1111/jcmm.13231

**Published:** 2017-06-13

**Authors:** Yanti Octavia, Georgios Kararigas, Martine de Boer, Ihsan Chrifi, Rinrada Kietadisorn, Melissa Swinnen, Hans Duimel, Fons K. Verheyen, Maarten M. Brandt, Daniela Fliegner, Caroline Cheng, Stefan Janssens, Dirk J. Duncker, An L. Moens

**Affiliations:** ^1^ Division of Experimental Cardiology Department of Cardiology, Thoraxcenter, Erasmus MC University Medical Center Rotterdam Rotterdam The Netherlands; ^2^ Department of Cardiology Cardiovascular Research Institute Maastricht Maastricht University Medical Centre Maastricht The Netherlands; ^3^ Institute of Gender in Medicine and Center for Cardiovascular Research Charite University Hospital Berlin Germany; ^4^ DZHK (German Centre for Cardiovascular Research) Berlin Germany; ^5^ Department of Cardiovascular Sciences University of Leuven Leuven Belgium; ^6^ Electron Microscopy Unit CRISP and Department of Molecular Cell Biology Maastricht University Medical Centre Maastricht The Netherlands

**Keywords:** anthracycline, antioxidant enzyme, cardiotoxicity, free radical, nitric oxide

## Abstract

The use of doxorubicin (DOXO) as a chemotherapeutic drug has been hampered by cardiotoxicity leading to cardiomyopathy and heart failure. Folic acid (FA) is a modulator of endothelial nitric oxide (NO) synthase (eNOS), which in turn is an important player in diseases associated with NO insufficiency or NOS dysregulation, such as pressure overload and myocardial infarction. However, the role of FA in DOXO‐induced cardiomyopathy is poorly understood. The aim of this study was to test the hypothesis that FA prevents DOXO‐induced cardiomyopathy by modulating eNOS and mitochondrial structure and function. Male C57BL/6 mice were randomized to a single dose of DOXO (20 mg/kg intraperitoneal) or sham. FA supplementation (10 mg/day per oral) was started 7 days before DOXO injection and continued thereafter. DOXO resulted in 70% mortality after 10 days, with the surviving mice demonstrating a 30% reduction in stroke volume compared with sham groups. Pre‐treatment with FA reduced mortality to 45% and improved stroke volume (both *P *<* *0.05 *versus* DOXO). These effects of FA were underlain by blunting of DOXO‐induced cardiomyocyte atrophy, apoptosis, interstitial fibrosis and impairment of mitochondrial function. Mechanistically, pre‐treatment with FA prevented DOXO‐induced increases in superoxide anion production by reducing the eNOS monomer:dimer ratio and eNOS S‐glutathionylation, and attenuated DOXO‐induced decreases in superoxide dismutase, eNOS phosphorylation and NO production. Enhancing eNOS function by restoring its coupling and subsequently reducing oxidative stress with FA may be a novel therapeutic approach to attenuate DOXO‐induced cardiomyopathy.

## Introduction

The use of DOXO, a member of the anthracycline family, as an effective broad spectrum chemotherapeutic drug has been hampered by its dose‐dependent cardiotoxicity, which leads to cardiomyopathy and heart failure 1. Approximately 5% of patients who receive 400–450 mg/m^2^ DOXO develop heart failure that may not be recognized for years 2. The mechanisms underlying DOXO‐induced cardiomyopathy are not completely understood, but may include direct effects on DNA, alteration of mitochondrial metabolism and reactive oxygen species (ROS) 3.

In the pathogenesis of DOXO‐induced myocardial dysfunction, the contribution of eNOS‐dependent oxidative stress has been reported 4. DOXO affects all NOS isoforms, causing the suppression of NOS activity, decreasing NO production and increasing superoxide anion generation 4. Cardioprotective effects of NO against DOXO‐induced cardiomyopathy have been previously reported 5. *In vivo* long‐term dietary nitrate supplementation in mice not only improved the left ventricular (LV) contractile and haemodynamic function, but also reduced the susceptibility to DOXO‐induced cell death 5. These findings implicate the importance of NO regulation as a key mediator in DOXO‐induced cardiomyopathy.

Several cofactors are required for NO generation, including tetrahydrobiopterin, oxygen and NADH. Reduced bioavailability of these factors could lead to NOS uncoupling and generate ROS instead of NO 6. FA, a member of the B‐vitamin family, can enhance NO generation in several ways, such as interacting directly or indirectly with eNOS 7. To this extent, a high dose of FA prevented ischaemia‐induced cardiac dysfunction and reduced reperfusion‐induced cardiac injury *via* eNOS recoupling 8.

In light of these considerations, we tested the hypothesis that FA effectively reduces DOXO‐induced cardiomyopathy in a mouse model of acute DOXO administration by modulating eNOS and preserving mitochondrial structure and function. In addition, we performed genome‐wide expression profiling to further identify potential factors implicated in DOXO‐induced cardiomyopathy.

## Materials and methods

### Cytotoxicity assay

HeLa and MDA‐MB‐231 cells (ATCC, Teddington, Middlesex, UK), a human cervical epithelial adenocarcinoma cell line and a human breast adenocarcinoma cell line, respectively, were maintained in Dulbecco's Modified Eagle Medium (ThermoFisher, Waltham, MA, USA) supplemented with 10% foetal bovine serum (ThermoFisher, Waltham, MA, USA) and 100U/ml penicillin‐streptomycin (ThermoFisher, Waltham, MA, USA). DOXO was added in a range of concentrations (10 μM to 0.1 nM) with/without FA ranging from 10 μM to 1 nM (supra‐ and physiological concentrations). Following this, the sulforhodamine B assay was performed, and the OD values were read at 24, 48 and 72 hrs.

### Experimental animals

Experiments were approved by the Institutional Animal Care and Use Committee and were carried out in accordance with the EU Directive 2010/63/EU for animal experiments. C57BL/6 mice (265 total, male, 9–11 weeks old; Charles River) were injected intraperitoneally with a single dose of DOXO hydrochloride (20 mg/kg; Pfizer, New York, NY, USA) as performed previously 9, 10. Soft‐dough diet (Transgenic Dough Diet; Bio‐Serv, Flemington, NJ, USA) with/without FA (10 mg/day; Sigma‐Aldrich, St.louis, MO, USA) was administered from 7 days before the DOXO injection until the end of the experiment (10 days).

### Echocardiographic measurements

Cardiac function and geometry were assessed by transthoracic echocardiography (Vevo 770) in anaesthetized mice (2.5% isoflurane) with body temperature maintained at 37°C, at baseline (before DOXO injection) and at 9 days post‐injection. LV internal dimensions at end‐diastole (LVIDd) and end‐systole (LVIDs), and LV posterior wall (LVPW) were measured from M‐mode images.

### Histological and ultrastructural examination of the heart

To assess myocyte width and fibrosis, hearts were fixed in 10% formalin, embedded in paraffin, cut in 5 μm sections and stained with haematoxylin–eosin (HE) and picrosirius red (PSR) using standard protocols as described previously 11. Cardiac cell death was detected with the TUNEL In Situ Cell Death Detection Kit (Roche Diagnostics, Almere, FL, The Netherlands) as previously described 12. In each group, 6–9 mice with 3–4 heart sections for each mouse were stained and analysed.

For electron microscopy, LV samples were fixed in 3% glutaraldehyde, 1.4% sucrose in 0.09 M potassium dihydrogen phosphate buffer (pH 7.4), post‐fixed in 1% osmium tetroxide in 0.1 M phosphate buffer (pH 7.4), dehydrated in graded ethanol series and embedded in Araldite. Ultrathin sections (40–60 nm) were stained with uranyl acetate and lead citrate. Photomicrographs were captured using a Philips CM100 transmission electron microscope (Philips 201).

### Mitochondrial function

Mitochondrial oxygen consumption was determined using a Clark electrode in a continuously stirred and sealed respiration chamber as previously described 13. Briefly, the hearts were excised, and skinned fibre preparations of the LV were used. Following the measurement of basal function, maximal ADP‐stimulated respiration was determined by exposing fibres to 2 mM ADP using glutamate (5 mM) and malate (2 mM) as substrates 13.

### Detection of superoxide anion

Superoxide anion generation was measured by two independent methods. Homogenized LV was evaluated by low concentration lucigenin‐enhanced chemiluminescence (5 μmol/l) using a single‐tube luminometer (Berthold FB12). As a measure of NOS‐derived superoxide anion generation, we evaluated the difference in superoxide anion generation after 20 min. incubation with N^G^‐ nitro‐L‐arginine methyl ester (L‐NAME: 100 μmol/l) and presented as ΔL‐NAME. Superoxide anion generation was also measured by dihydroethidium (DHE) staining of LV tissue sections.

### Protein measurements

Protein levels were determined in homogenates of myocardial samples as previously described 14. Primary antibodies were phospho‐eNOS S1177, eNOS, nNOS, iNOS, GAPDH (Cell Signaling, Danvers, MA, USA; BD Transduction Laboratories, Heidelberg, Germany; Santa Cruz, Abingdon, UK; Imgenex, Cambridge, UK) and used at 1:1000.

Monomer:dimer ratio was determined using cold SDS‐PAGE Western blot in 4%–7% SDS‐Tris gels at 4°C. Subsequently, the proteins were transferred to nitrocellulose membranes and were probed with primary and secondary antibodies (LI‐COR). All blots were analysed using the Odyssey system.

S‐glutathionylation of eNOS was determined by co‐immunoprecipitation of LV homogenates. Briefly, protein G (Invitrogen)‐conjugated anti‐eNOS (Santa Cruz) was incubated overnight with LV homogenates at 4°C. After washing with PBS, eNOS was immunoblotted with anti‐GSH (Virogen, Watertown, MA, USA) in the presence or absence of DTT (10 μmol/l) to remove the glutathionylation modification of eNOS.

### Myocardial NO production

Myocardial NO production was evaluated as the measurement of nitrate + nitrite using the Griess reaction colorimetric assay (Cayman CHemical, Ann Arbor, MI, USA) per manufacturer's instructions.

### Antioxidant activity

Total superoxide dismutase (SOD) activity and the GSSG (oxidized glutathione)‐to‐GSH (reduced glutathione) ratio were assessed with commercially available kits (Cayman Chemical, Ann Arbor, MI, USA) per manufacturer's instructions.

### Hybridization and microarray profiling

Total RNA was isolated using the RNeasy Fibrous Tissue Mini kit (Qiagen, Venlo, NB, The Netherlands) per manufacturer's instructions. RNA quality and quantity were established using a 2100 Bioanalyzer (Agilent Technologies, Santa Clara, CA, USA). Biotinylated complementary RNA was prepared and hybridized to the Mouse Gene 1.0 ST array (Affymetrix, Waltham, MA, USA) according to the Affymetrix processing protocol. The array was scanned in a GeneChip Scanner 3000. The quality of hybridization was assessed in all samples following manufacturer's recommendations. Microarray data are deposited in the Gene Expression Omnibus database (accession No. GSE64476). The computational and statistical analysis of the microarray data was carried out using the R version 2.14.2 software (https://www.r-project.org/) and the Bioconductor packages (https://www.bioconductor.org/) as performed previously 11, 15, 16, 17. Gene lists were generated selecting for candidates with an uncorrected *P* value threshold of 0.01 as performed recently 15.

### Quantitative real‐time RT‐PCR

Quantitative analysis was performed by real‐time PCR with specific primers and SensiMix™ SYBR^®^ & Fluorescein Kit using iQ5 (Bio‐Rad, Veenendaal, UT, The Netherlands). Primer sequences are shown in Table S1.

### Statistical analysis

Data are presented as mean ± S.E.M. Analysis of survival rates was performed with the log‐rank test. All data were tested using two‐way anova with Student‐Newman‐Keuls post hoc test considering *P *<* *0.05 significant.

## Results

### FA did not interfere with DOXO cell‐killing capacity

To investigate whether treatment with FA reduces the main tumour cell‐killing effects of DOXO, we employed the human cervical epithelial adenocarcinoma HeLa cell line and the human breast adenocarcinoma MDA‐MB‐231 cell line treating them with different doses of DOXO and FA. DOXO in the absence or presence of FA at various concentrations exerted similar cytotoxic effects in HeLa and MDA‐MB‐231 cancer cells (Fig. S1). Therefore, the antitumour activity of DOXO was not affected by FA.

### FA blunted DOXO‐induced mortality

**Figure 1:**
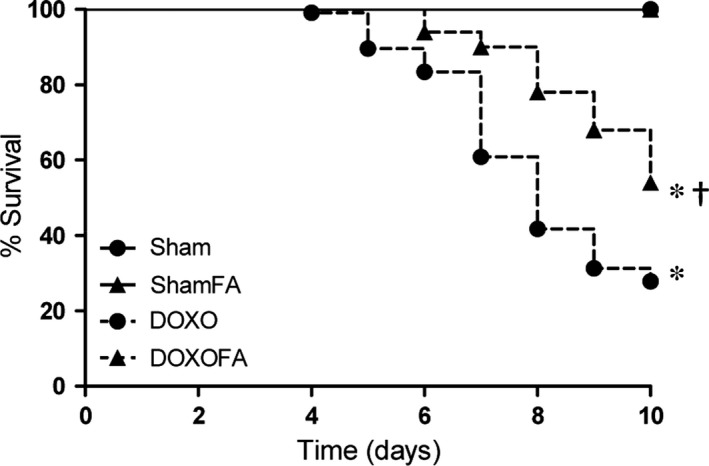
Effects on mortality. DOXO, doxorubicin; FA, folic acid. *P<0.01 *versus* corresponding Sham; †*P*<0.01 DOXOFA *versus* DOXO.

DOXO resulted in 70% mortality 10 days after injection, which was decreased to 45% by pre‐treatment with FA (Fig. 1). At baseline, body weights were similar in all groups. DOXO produced a significant loss of body weight *versus* the corresponding sham group, which was not significantly affected by FA (Table S2).

### FA normalized DOXO‐induced left ventricular structural and functional changes

**Figure 2:**
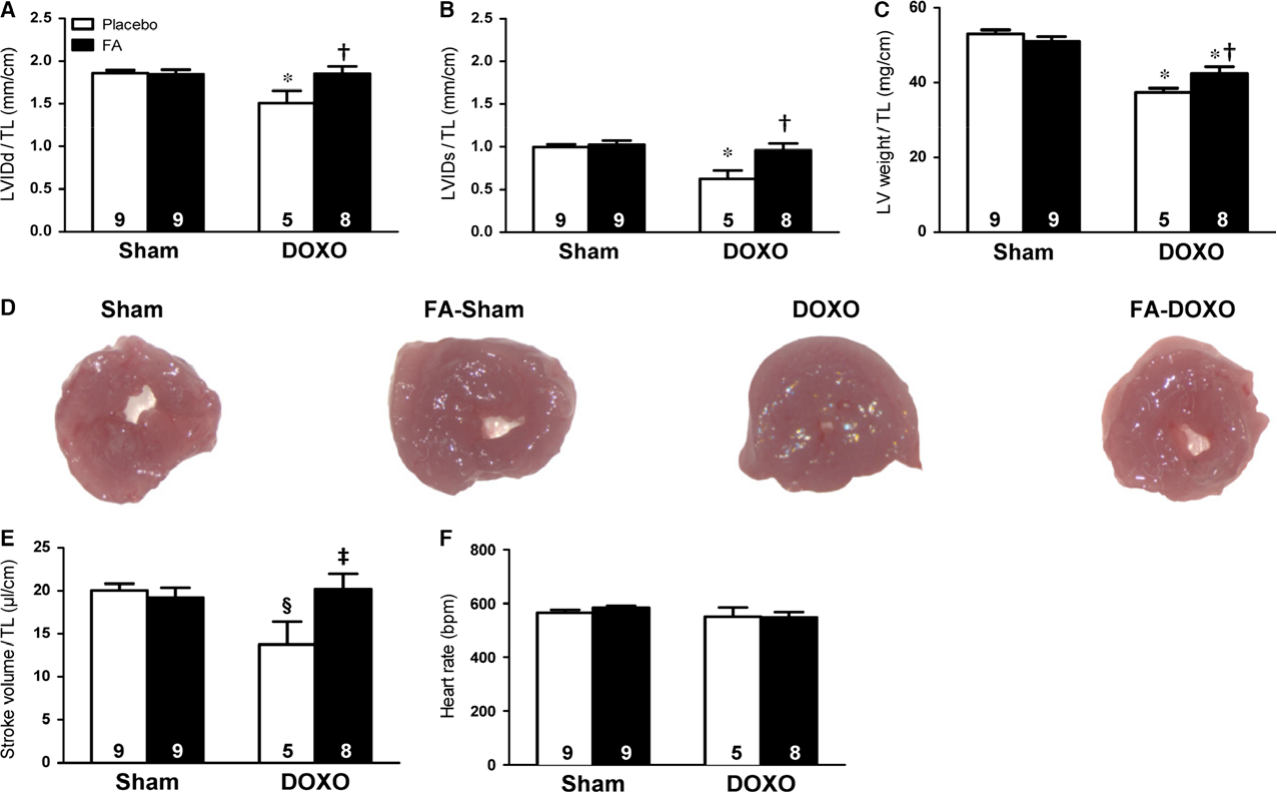
Effects on cardiac function and structure. Left ventricular (LV) internal diameter at end‐diastole (LVIDd), (**A**) and end‐systole (LVIDs), (**B**) normalized to tibia length (TL). (**C**) LV weight/TL. (**D**) LV lumen. (**E**) Stroke volume. (**F**) Heart rate. The *n* is indicated directly in the graph bars in all figures. BPM, beats per min. *P <* *0.01 *versus* corresponding Sham; §*P *<* *0.05 *versus* corresponding Sham; †*P *<* *0.01 FA‐DOXO *versus* DOXO; ‡*P *<* *0.05 FA‐DOXO *versus* DOXO.

DOXO led to significant reductions in LVIDd and LVIDs, LV weight and LV lumen (Fig. 2A–D). These DOXO‐induced structural changes had the functional consequence of decreased stroke volume *versus* the corresponding sham group (Fig. 2E). Importantly, pre‐treatment with FA attenuated all these deleterious changes (Fig. 2A–E). Heart rate was not significantly affected in any group (Fig. 2F).

### FA attenuated DOXO‐induced cardiomyocyte atrophy, cardiac apoptosis and fibrosis

**Figure 3:**
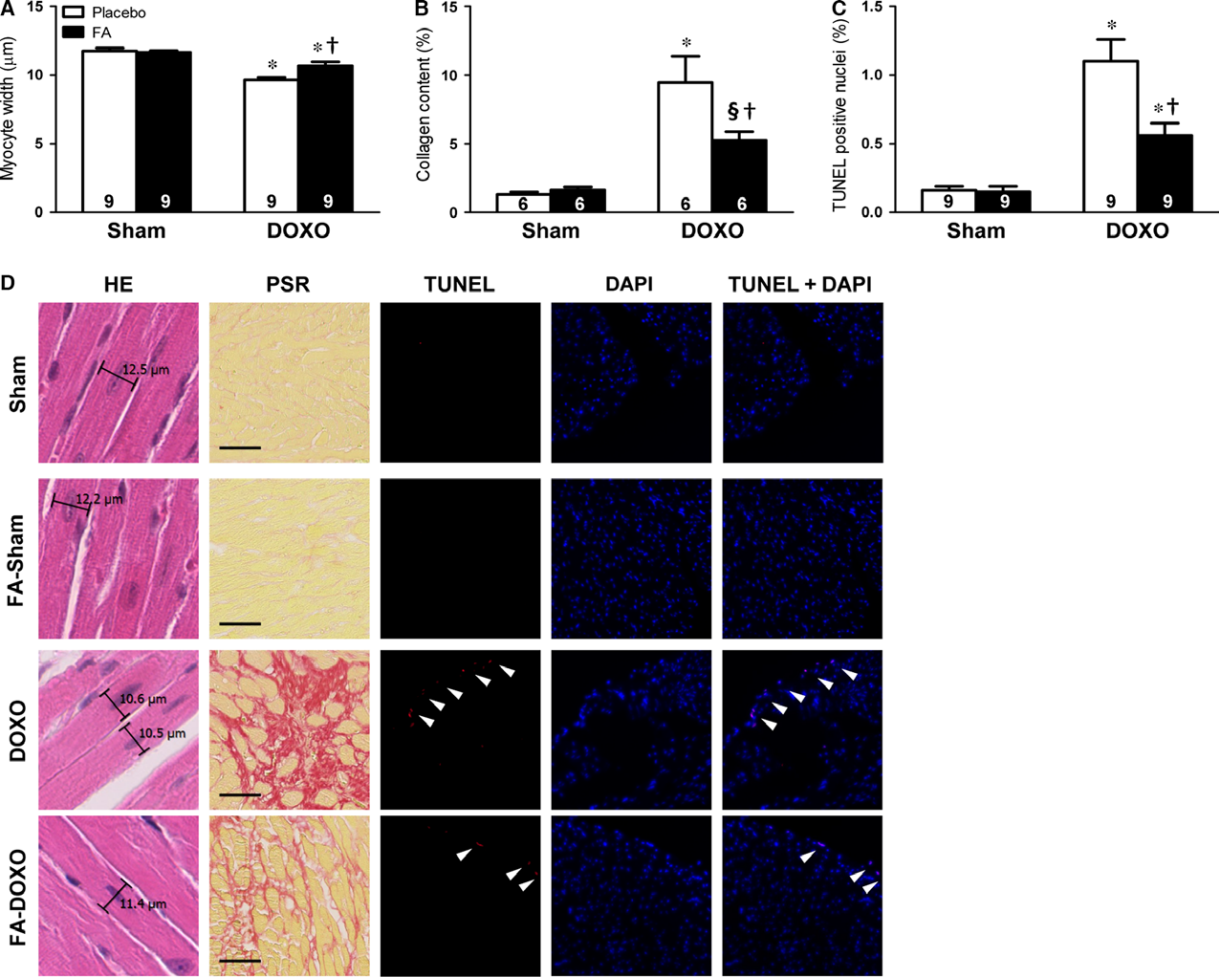
Effects on cardiomyocyte width, cardiac fibrosis and apoptosis. (**A**) Myocyte width evaluation using haematoxylin–eosin (HE) staining. (**B**) Collagen content measurements using picrosirius red (PSR) staining. (**C**) Cardiomyocyte apoptosis as assessed by TUNEL positive nuclei. (**D**) Representative images of stained sections. *P <* *0.01 *versus* corresponding Sham; §*P *<* *0.05 *versus* corresponding Sham; †*P *<* *0.01 FA‐DOXO *versus* DOXO.

Investigating the underlying cellular mechanisms, we found that the LV myocardium of mice receiving DOXO alone displayed a reduction in cardiomyocyte width and increases in collagen content and apoptosis (Fig. 3A–D). FA exerted significant protective effects against these DOXO‐induced deleterious effects (Fig. 3A–D).

### FA prevented DOXO‐dependent impairment of mitochondrial function and ultrastructure

**Figure 4:**
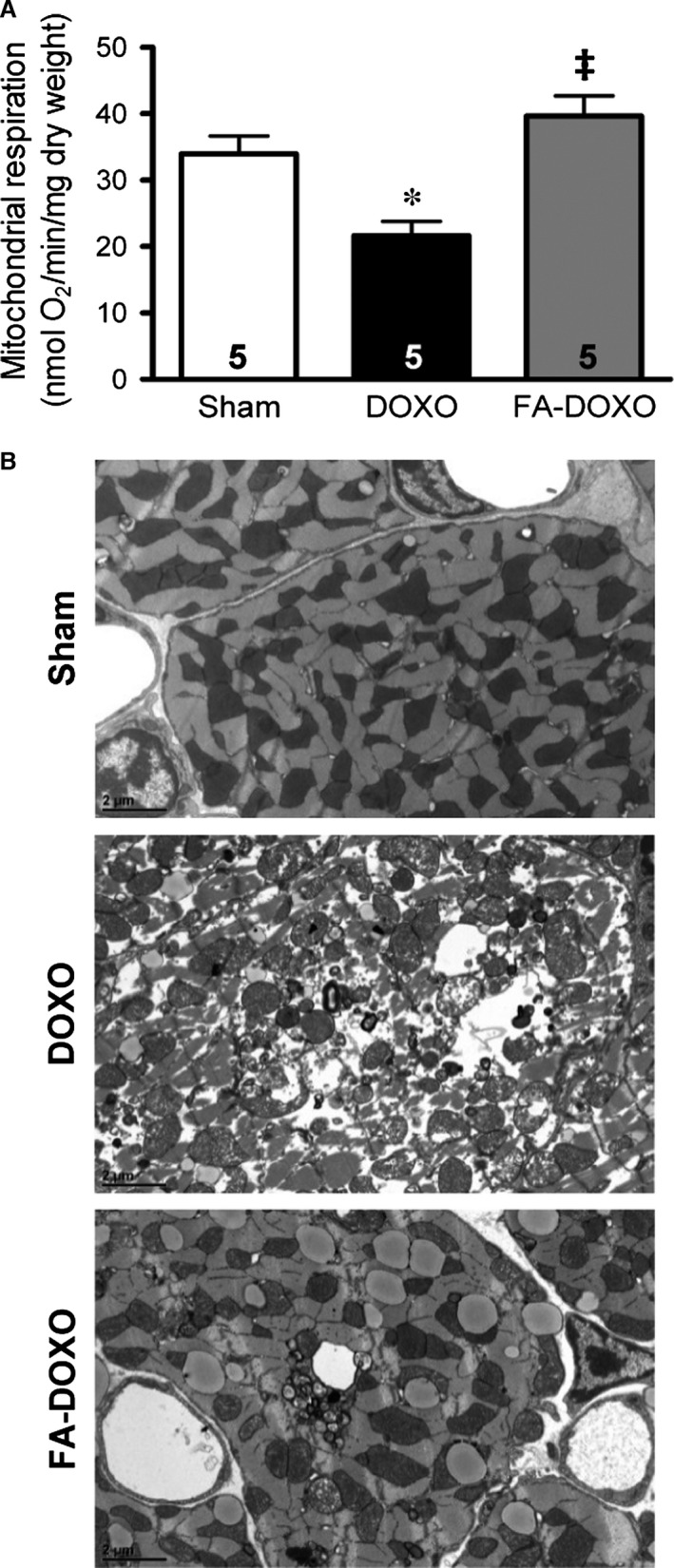
Effects on mitochondrial function and ultrastructure. (**A**) Mitochondrial oxygen consumption as measured by state 3. (**B**) Representative transmission electron microscopy images. *P <* *0.01 *versus* Sham; ‡*P *<* *0.05 *versus* DOXO.

DOXO has been shown to inhibit complex I of the electron transport chain and concomitantly induces mitochondrial dysfunction, while nitrate supplementation has been shown to protect mitochondrial oxygen respiration 5. Therefore, we hypothesized that FA, as NOS modulator, might exert further protective effects against mitochondrial dysfunction. As in our preliminary measurements we did not find any significant differences between the sham and FA‐sham groups, we merged the two groups into a single one to lower the number of animals used. We found that while ADP‐stimulated mitochondrial respiration (state 3) was diminished in the DOXO group, it was maintained in mice pre‐treated with FA (Fig. 4A).

Ultrastructural analysis of the sham group showed normal mitochondrial structure with preserved internal architecture and surrounding organized myofibrils (Fig. 4B). Samples of DOXO mice demonstrated prominent patchy myofibrillar loss and disarray. Moreover, mitochondria showed significant abnormalities, such as enlargement, contour irregularities, increased spacing between cristae and disruption of the internal architecture. Pre‐treatment with FA markedly prevented DOXO‐induced mitochondrial ultrastructural degeneration (Fig. 4B).

### FA blunted DOXO‐induced oxidative stress, eNOS and antioxidant dysregulation

**Figure 5:**
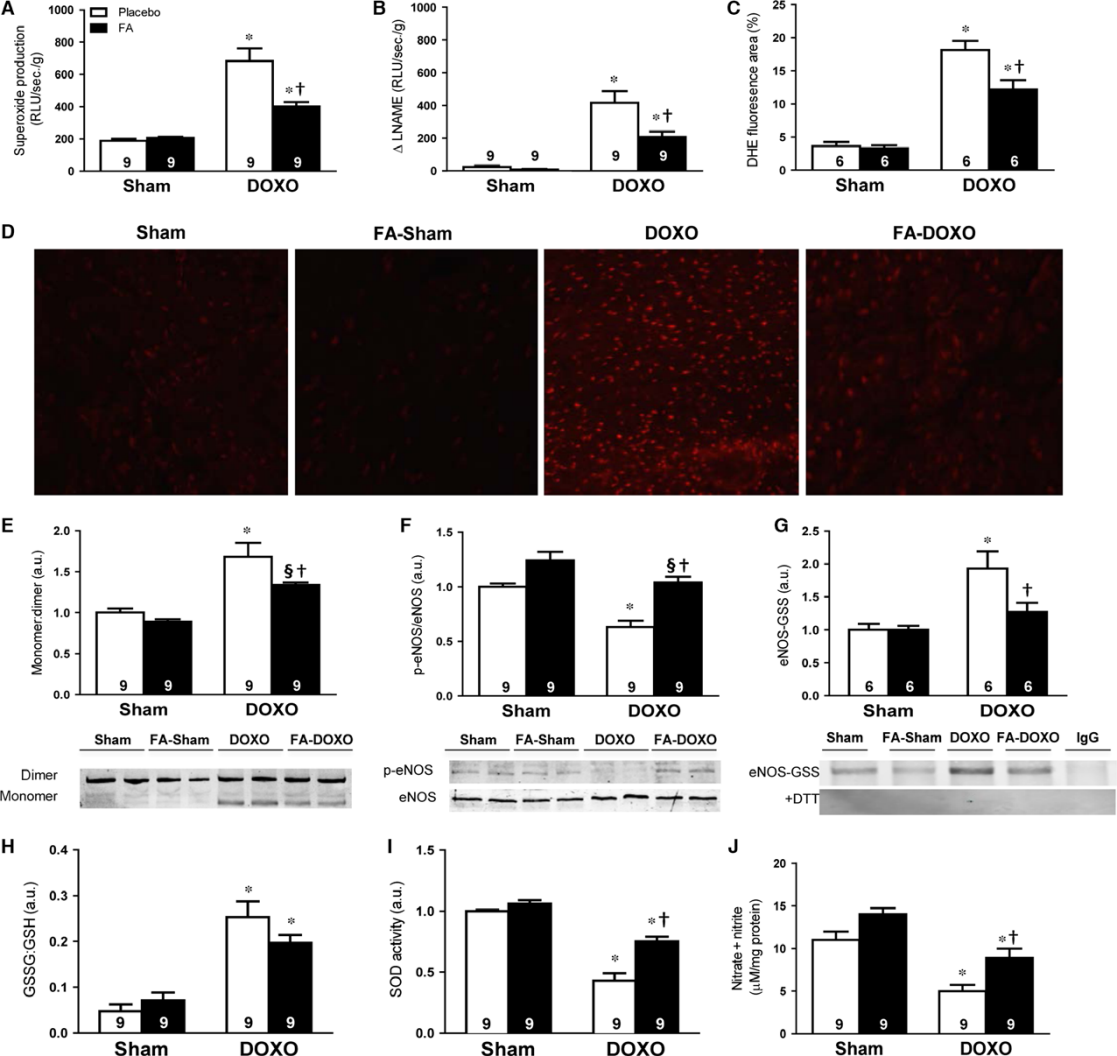
Effects on oxidative stress, endothelial nitric oxide synthase (eNOS) modulation and antioxidant system. (**A**) Using lucigenin‐enhanced chemiluminescence, superoxide anion production is expressed as the relative light unit (RLU) per second per gram of protein. (**B**) Substraction of lucigenin‐enhanced chemiluminescence with N^G^‐ nitro‐L‐arginine methyl ester (L‐NAME) incubation from total superoxide anion, presented as Δ L‐NAME. (**C**) Dihydroethidium (DHE) fluorescence quantification from LV sections. (**D**) Representative DHE‐stained sections. (**E**) Monomer:dimer ratio of eNOS. (**F**) Phosphorylation of eNOS at serine 1177. (**G**) S‐glutathionylation of eNOS. The representative co‐immunoprecipitation of eNOS without (top) and with (bottom) DTT to remove glutathionylation. Unspecific mouse IgG antibody was used as a negative control. (**H**) Ratio between oxidized glutathion (GSSG) and reduced glutathion (GSH). (**I**) Superoxide dismutase (SOD) activity. (**J**) Cardiac NO production analysed by total nitrate and nitrite. *P <* *0.01 *versus* corresponding Sham; §*P *<* *0.05 *versus* corresponding Sham; †*P *<* *0.01 FA‐DOXO *versus* DOXO.

To unravel the molecular underpinnings of the FA‐mediated protection against the deleterious effects of DOXO, we studied the effects of FA on DOXO‐induced oxidative stress. Indeed, superoxide anion production was significantly increased in LV homogenates from DOXO mice *versus* sham mice, which appeared to be largely NOS‐dependent, as it was markedly attenuated by the NOS inhibitor L‐NAME (Fig. 5A‐D). DOXO did not lead to changes in nNOS and iNOS levels or the respective monomer:dimer ratios (Fig. S2). However, DOXO increased the eNOS monomer:dimer ratio, indicative of eNOS uncoupling, and decreased eNOS phosphorylation at S1177, indicative of reduced eNOS activity (Fig. 5E and F).

S‐glutathionylation of eNOS has been recently found as the trigger of eNOS uncoupling 18. Therefore, we assessed the levels of eNOS S‐glutathionylation and found a significant increase related to DOXO *versus* sham (Fig. 5G*)*. S‐glutathionylation occurs due to elevation of glutathione disulphide (GSSG, oxidized glutathione) 18. The ratio GSSG to glutathione (GSH, reduced glutathione) was increased in DOXO *versus* sham group (Fig. 5H). In parallel, more than 50% superoxide dismutase (SOD) activity was decreased (Fig. 5I). DOXO reduced myocardial NO production by 50% *versus* sham mice (Fig. 5J).

Notably, pre‐treatment with FA restored eNOS and antioxidant enzymes dysregulation, which were associated with an increase in NO production and a decrease in NOS‐dependent superoxide anion formation (Fig. 5A–J).

### FA modulated DOXO‐induced transcriptomic changes

To further investigate the molecular mechanisms of FA‐mediated protection against DOXO‐induced cardiomyopathy, we performed genome‐wide expression profiling of LV samples. To extract biologically useful information, we examined those genes with an unadjusted *P *<* *0.01 as performed previously 15. Our analysis revealed that 152 transcript clusters were regulated by DOXO alone *versus* the sham group (Table S3), while 68 transcript clusters were differentially regulated between the FA‐DOXO and DOXO groups (Table S4). Interestingly, 71 transcript clusters were found significant by the interaction analysis (Table S5). Among the most up‐regulated genes by DOXO, we identified a ca. 10‐fold increase in metallothionein‐2 (*Mt2*), a gene that is involved in oxidative stress, and apoptotic factor lipocalin‐2 (*Lcn2*) with a ca. fivefold increase compared with controls. Furthermore, we found that the desmosomal gene *Adam4* was repressed in DOXO‐treated mice by ca. 30%, while its expression was restored in FA‐DOXO‐treated mice. Also, several members of the cysteine protease inhibitors (stefins) were induced in DOXO‐treated mice *versus* controls, while their expression was normalized by FA.

**Figure 6:**
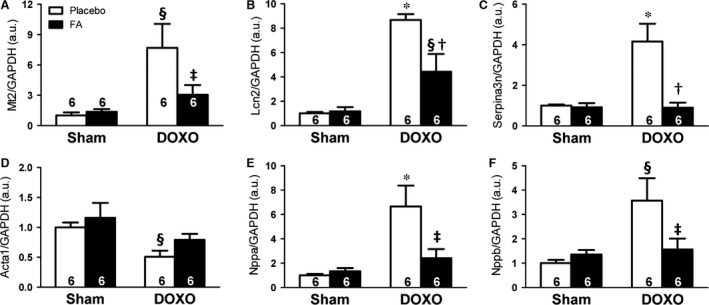
Effects on transcriptomic regulation. Messenger RNA levels of *Mt2* (**A**), *Lcn2* (**B**), *Serpina3n* (**C**), *Acta1*(**D**), *Nppa* (**E**), *Nppb* (**F**) normalized to *Gapdh*. *P <* *0.01 *versus* corresponding Sham; §*P *<* *0.05 *versus* corresponding Sham; †*P *<* *0.01 FA‐DOXO *versus* DOXO; ‡*P *<* *0.05 FA‐DOXO *versus* DOXO.

To validate genes involved in DOXO‐induced cardiomyopathy and modulated by FA, we performed quantitative real‐time RT‐PCR analysis selecting several candidates that exhibited significant induction. Similar to the microarray data analysis, we found that DOXO induced the expression of *Mt2, Lcn2,* and *Serpina3n*, which was decreased by FA (Fig. 6A–C). We further analysed the expression of foetal genes and found a decrease in *Acta1* and an increase in *Nppa* and *Nppb* expression in the DOXO group, which was normalized in the FA‐DOXO group (Fig. 6D–F).

## Discussion

In a mouse model of DOXO‐induced cardiomyopathy, we report for the first time that pre‐treatment with FA, an eNOS modulator, attenuated DOXO‐induced mortality and markedly reduced LV structural damage. Oral supplementation of FA attenuated cardiomyocyte atrophy, cardiac apoptosis and fibrosis, maintained mitochondrial integrity and reduced superoxide anion production. Mechanistically, our data suggest that these cardioprotective effects of FA may be mediated *via* eNOS recoupling, with enhanced phosphorylation of eNOS at S1177 and reduced glutathionylation of eNOS, both of which result in improved cardiac NO production.

Anthracycline‐induced cardiomyopathy remains a significant problem in both oncology and cardiology practices 2. Our hypothesis to test a pharmacological intervention with FA against DOXO‐induced cardiomyopathy was based on the ability of FA to improve outcome in primary prevention of cardiovascular disease 7, 8. Our data demonstrate that treatment with FA significantly reduced DOXO‐induced mortality. This raised the question whether FA could actually interfere with and attenuate the antitumour capacity of DOXO. Our *in vitro* data showed that co‐administration of DOXO and FA to the tumour cell lines HeLa and MDA‐MB‐231 did not interfere with DOXO main effects, as DOXO was still able to exert its antitumour actions.

The effects of DOXO on LV remodelling and dysfunction are still incompletely understood, as several studies in rodents have demonstrated contradictory results depending on the dose and duration following DOXO administration. Consequently, DOXO has been reported to elicit inward remodelling 9, 19, outward remodelling 20, 21 or no effects on cardiac remodelling 22, 23, irrespective of the observed reductions in stroke volume 19, 23, 24 and cardiac output 25. In our model, DOXO induced inward LV remodelling, as indicated by the reduced cardiac dimensions resulting in decreased stroke volume. However, the pre‐treatment with FA led to a significant reduction in DOXO‐induced cardiomyopathy, and cardiac morphometry and function were normalized back to sham levels.

Consistent with previous observations 20, 21, we observed that interstitial fibrosis and apoptosis are prominent features of DOXO‐induced cardiomyopathy and likely contributed to the effects of DOXO on LV dysfunction. FA not only attenuated these pathological changes, but also reduced DOXO‐induced cardiomyocyte atrophy, thereby blunting the loss of LV mass.

Furthermore, it was demonstrated that DOXO results in desmin disruption and myofibrillar disorganization 26. As desmin adheres to mitochondria, desmin disarray might be related with mitochondrial dysfunction. Along this line, previous studies have suggested the involvement of mitochondria in DOXO‐induced cardiomyopathy 5, 26. In the present study, we confirmed DOXO‐induced mitochondrial dysfunction, as indicated by impaired mitochondrial function and major mitochondrial morphological changes. Notably, FA was able to normalize mitochondrial function and to maintain mitochondrial integrity.

FA regulates intracellular eNOS signalling *via* indirect or direct mechanisms, including eNOS phosphorylation at multiple residues 7, leading to NO release and restoring the balance between NO and superoxide anion production, which is associated with cardioprotection. However, the role of eNOS in DOXO‐induced cardiomyopathy remains incompletely understood. It was reported that DOXO generates superoxide anion, which was decreased in eNOS KO mice and increased in mice with overexpression of eNOS 27. These results suggested that DOXO induces superoxide anion *via* an eNOS‐dependent mechanism, and removal of dysfunctional eNOS is beneficial against DOXO‐induced cardiomyopathy. However, the authors did not find eNOS uncoupling at day 5 in WT mice, while we found eNOS uncoupling at day 10. Importantly, we found that pre‐treatment with FA prevented eNOS uncoupling and normalized the phosphorylation of eNOS at S1177, resulting in decreased superoxide anion generation and restored myocardial NO production, thereby leading to an adaptive response preserving cardiac function, ultimately providing cardioprotection and improving mortality. Therefore, it would be very informative to obtain data on eNOS uncoupling on a later time‐point or in mice overexpressing eNOS, which could be compared with the data presented here.

We also found a significantly decreased total LV SOD activity in DOXO mice, which was blunted by pre‐treatment with FA. In addition, S‐glutathionylation of eNOS, which can be a direct result of elevated GSSG levels or an indirect result of the formation of unstable protein radicals, may adversely modulate eNOS structure and function and generate superoxide anion 18. Thus, it is important to understand the effect of this oxidative modification and modulate S‐glutathionylation. Interestingly, we found that DOXO increased the GSSG:GSH ratio, leading to S‐glutathionylation of eNOS, and that this was blunted by pre‐treatment with FA.

There are other mechanisms by which DOXO can lead to altered cardiac metabolism and induce superoxide anion generation 3. These include DOXO‐induced ROS generation through topoisomerase II‐dependent DNA double‐strand breaks, which lead to apoptosis and mitochondrial damage 28. Recently, a novel mechanism on the involvement of late sodium current (*I*_Na_) in the modulation of cardiac redox balance in DOXO‐induced cardiomyopathy was reported 29. Interestingly, it was shown that ranolazine, an *I*_Na_ inhibitor, protects against DOXO‐induced cardiac dysfunction and remodelling 29. Furthermore, both DOXO and FA have been shown to produce alterations in DNA methylation status in the heart 30, 31. On the basis of these observations, it is possible that other effects of FA may have also contributed to the protection against DOXO‐induced toxicity.

The molecular signalling pathways linking ROS with gene modifications involved in pathophysiological remodelling of the heart are not fully understood. Therefore, we performed comparative transcriptomic analysis, which revealed marked DOXO‐induced up‐regulation of *Nppa* and *Nppb*, which are markers of cardiac dysfunction, and down‐regulation of the desmosomal gene *Adam4* and the cysteine protease inhibitors stefins. Desmosomes are crucial for tissue homoeostasis and cardiac integrity 32. The cysteine protease inhibitors stefins have been proposed as prognostic and diagnostic tools for cancer and may have an important role in desmosome‐mediated cell–cell adhesion 33. The role of this regulatory family in the heart is unknown and warrants further investigation. Although in the present study we did not measure protein abundance or activity, we put forward that these may be additional mechanisms involved in the protective effects of FA against DOXO‐induced cardiomyopathy.

Our study was limited to a single dose of acute DOXO administration in mice in the absence of cancer, while in patients DOXO is chronically administered. However, this is an established model of DOXO‐induced cardiomyopathy that shares features with the cardiomyopathy observed in patients following chronic administration of DOXO 9, 10, 27, and our *in vitro* data showed that co‐administration of FA did not affect the tumour cell‐killing capacity of DOXO. Nevertheless, further experiments are necessary to investigate the effects of FA in a chronic model of DOXO administration, which may reflect the clinical situation more closely. Moreover, in our study, mortality reached 70% after 10 days of DOXO administration, which might be considered relatively high. However, 50% mortality was previously reported after 8 days of DOXO administration 27, at which point we observed 60% mortality, and even 90% mortality after 10 days of DOXO administration has been reported 10. It has to be noted, however, that this might be a strain‐specific effect, as ca. 21% mortality was reported after 9 days of DOXO administration in CF‐1 outbred mice 34. Furthermore, we limited ourselves to a single dose of FA, which does not exclude varying effects at different doses. Nevertheless, our study indicates that FA decreased acute DOXO‐induced cardiomyopathy and mortality even at supratherapeutic levels of DOXO. Considering the sex differences in DOXO cardiotoxicity recently reported 35, it would also be interesting to assess whether the efficacy of FA to attenuate DOXO‐induced cardiomyopathy might differ between the sexes.

**Figure 7:**
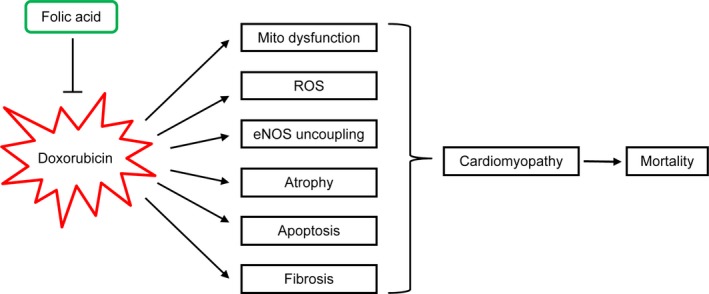
Schematic presentation of the prevention of DOXO‐induced cardiomyopathy and mortality by FA.

In conclusion, the development of therapeutic strategies targeting eNOS *via* FA supplementation may prove a major step forward in addressing DOXO‐induced cardiomyopathy. Considering its robust safety profile and its widespread use as dietary supplement, FA may hold promise for oncologists and haematologists to prevent DOXO‐induced cardiomyopathy and mortality (Fig. 7).

## Conflict of interest

The authors confirm that there is no conflict of interest.

## Funding source

This work was supported by the Dutch Heart Foundation (NHS project number 30982277) and NWO/ZonMw (Vidi/Aspasia‐funding).

## Author contribution

YO, ALM and DJD conceived the study. YO, GK and ALM designed the study. YO, GK, MdB, RK, MS, HD, FKV, HD, IC and MMB acquired the data. Yo, GK, MdB, HD, FKV, DF, CC and SJ analysed and interpreted the data. YO, GK and DJD drafted the manuscript. All authors critically revised the manuscript for important intellectual content and approved the manuscript for submission.

## Supporting information


**Figure S1** Folic acid (FA) effects on doxorubicin (DOXO)‐treated HeLa and MDA‐MB‐231 cells
**Figure S2** Neuronal and inducible nitric oxide synthase (nNOS and iNOS, respectively) protein levels normalized to GAPDH and monomer (m):dimer (d)
**Table S1** Primer sequences for real‐time RT‐PCR
**Table S2** Anatomical data
**Table S3** DOXO‐regulated probe sets
**Table S4** FA effects on gene expression after DOXO injection
**Table S5** Interaction analysis of sham, DOXO and DOXO with FA on gene expression
