# Supplementary material for: Folic acid reduces doxorubicin‐induced cardiomyopathy by modulating endothelial nitric oxide synthase
Source: J Cell Mol Med. 2017 Jun 13;21(12):3277–87. doi: 10.1111/jcmm.13231 (PMC5706529; doi:10.1111/jcmm.13231)
Supplement: Supplementary file 1 — Figure S1 Folic acid (FA) effects on doxorubicin (DOXO)‐treated HeLa and MDA‐MB‐231 cells Figure S2 Neuronal and inducible nitric oxide synthase (nNOS and iNOS, respectively) protein levels normalized to GAPDH and monomer (m):dimer (d) Table S1 Primer sequences for real‐time RT‐PCR Table S2 Anatomical data Table S3 DOXO‐regulated probe sets Table S4 FA effects on gene expression after DOXO injection Table S5 Interaction analysis of sham, DOXO and DOXO with FA on gene expression [file JCMM-21-3277-s001.pdf]

Supplementary data

Folic acid reduces doxorubicin-induced cardiomyopathy by modulating endothelial nitric oxide synthase

Yanti Octavia [a, b, #], Georgios Kararigas [c, d, #, *], Martine de Boer [a], Ihsan Chrifi [a], Rinrada Kietadisorn [b], Melissa Swinnen [e], Hans Duimel [f], Fons K Verheyen [f], Maarten M Brandt [a], Daniela Fliegner [c], Caroline Cheng [a], Stefan Janssens [e], Dirk J Duncker [a, $, *], An L Moens [a, b, $]

[a] Division of Experimental Cardiology, Department of Cardiology, Thoraxcenter, Erasmus MC, University Medical Center Rotterdam, Rotterdam, The Netherlands

[b] Department of Cardiology, Cardiovascular Research Institute Maastricht, Maastricht University Medical Centre, Maastricht, The Netherlands

[c] Institute of Gender in Medicine and Center for Cardiovascular Research, Charite University Hospital, Berlin, Germany

[d] DZHK (German Centre for Cardiovascular Research), partner site Berlin, Germany

[e] Department of Cardiovascular Sciences, University of Leuven, Leuven, Belgium

[f] Electron Microscopy Unit, CRISP and Department of Molecular Cell Biology, Maastricht University Medical Centre, Maastricht, The Netherlands

[#] Y.O. and G.K. contributed equally to this paper.

[$] D.J.D. and A.L.M. contributed equally to this paper.

***Corresponding Author** Prof. Georgios Kararigas, Institute of Gender in Medicine & Center for Cardiovascular Research, Charite University Hospital, Hessische Str. 3-4, 10115 Berlin, Germany. Tel: +49-30-450525355; Fax: +49-30-450525943; Email: george.kararigas@charite.de **or**

Prof. Dirk J Duncker, Division of Experimental Cardiology, Department of. Cardiology, Thoraxcenter, Erasmus University Medical Center, PO Box 2040, 3000 CA Rotterdam, The Netherlands. Tel: +31107030866; Fax: +31107044769; Email: d.duncker@erasmusmc.nl

Figure S1 Folic acid (FA) effects on doxorubicin (DOXO)-treated HeLa and MDA-MB-231 cells

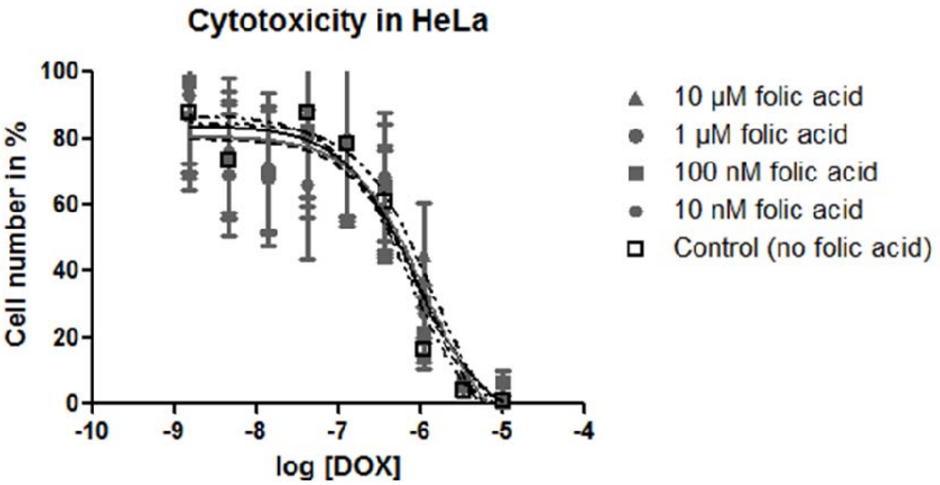

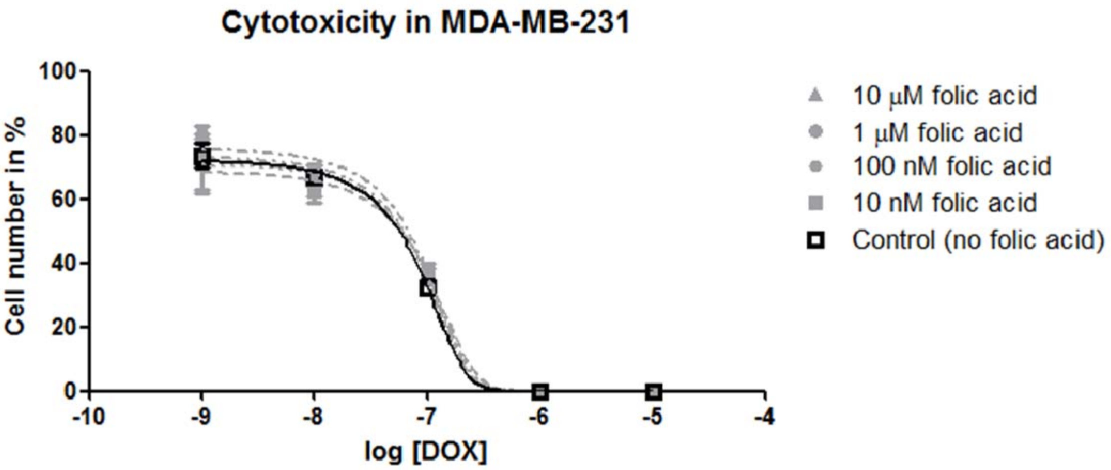

Figure S2 Neuronal and inducible nitric oxide synthase (nNOS and iNOS, respectively) protein levels normalized to GAPDH and monomer (m):dimer (d)

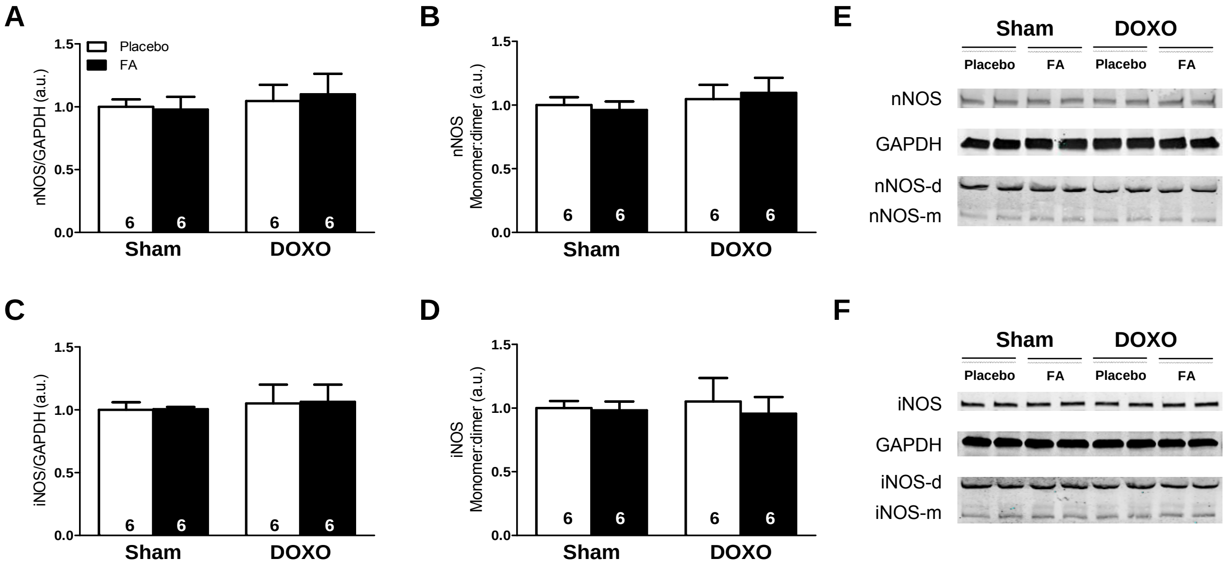

Table S1 Primer sequences for real-time RT-PCR

**Gene**	**Primer sense**	**Primer antisense**
Metallothionin 2	CTCGTCGATCTTCAACCGCC	CGGAAGCCTCTTTGCAGATG
Lipocalin 2	CCAGCCACCATACCAAGGAG	GACGCCATTGGTGGTGTTAAG
Serine (or cysteine) peptidase inhibitor, clade A, member 3N (Serpina3n)	TCTTCTCCACACAGGCTGAC	CAGAGGGTACAGTTTCGCAGA
α-skeletal actin	TGACGTGTACATAGATTGACTCGTTT	TGGCTGGCTTTAATGCTTCA
Atrial natriuretic peptide	TTCCTCGTCTTGGCCTTTTG	CCTCATCTTCTACCGGCATCTTC
Brain natriuretic peptide	GGGAGAACACGGCATCATTG	ACAGCACCTTCAGGAGATCCA

Table S2 Anatomical data

		Sham	Doxorubicin	
Body weight baseline (g)	Placebo	26.8 ± 0.6	26.6 ± 0.3	
	FA	27.1 ± 0.3	27.7 ± 0.4	
Body weight follow up (g)	Placebo	27.6 ± 0.4	22.1 ± 0.5	*
	FA	27.0 ± 0.5	22.5 ± 0.5	*
Tibia length (cm)	Placebo	1.80 ± 0.01	1.78 ± 0.01	*
	FA	1.79 ± 0.01	1.78 ± 0.01	
LV weight (mg)	Placebo	95.5 ± 1.7	65.7 ± 2.0	*
	FA	91.4 ± 2.3	74.5 ± 3.8	*†
RV weight (mg)	Placebo	25.0 ± 0.7	17.9 ± 0.9	*
	FA	25.6 ± 1.0	20.2 ± 1.2	*
LV weight/tibia length (mg/cm)	Placebo	53.1 ± 1.0	37.4 ± 1.1	*
	FA	51.0 ± 1.3	42.5 ± 1.7	*†
LVIDd (mm)	Placebo	3.35 ± 0.06	2.66 ± 0.25	*
	FA	3.31 ± 0.09	3.31 ± 0.15	†
LVIDs (mm)	Placebo	1.79 ± 0.07	1.10 ± 0.17	*
	FA	1.83 ± 0.09	1.71 ± 0.15	†
Stroke volume (μl)	Placebo	36.1 ± 1.4	24.2 ± 4.6	*
	FA	34.4 ± 2.1	36.0 ± 3.2	†
LVPWd (mm)	Placebo	0.96 ± 0.02	1.00 ± 0.17	
	FA	0.94 ± 0.04	0.88 ± 0.02	

Data are presented as mean ± SEM. LV, left ventricle; RV, right ventricle; LVIDd, LV internal dimensions at end-diastole; LVIDs, LVID at end-systole; LVPWd, LV posterior wall at end diastole. *P<0.05 vs. Sham; †P<0.05 vs. DOXO

Table S3 Doxorubicin-regulated probe sets

Affymetrix ID	Gene symbol	Gene name	*P* value	Fold change relative to DOXO
10566258			0	0.06
10598032			0	0.16
10593058			0	1.56
10566254	Hbb-b1	hemoglobin, beta adult major chain	0	0.38
10574023	Mt2	metallothionein 2	0	10.13
10342598			0	1.57
10598053			0	0.31
10342849			0	1.40
10604877	Mir465	microRNA 465	0	1.37
10342788			0.001	1.41
10341762			0.001	1.45
10338956			0.001	1.55
10342109			0.001	0.56
10444298	H2-Eb1	histocompatibility 2, class II antigen E beta	0.001	0.62
10493831	S100a8	S100 calcium binding protein A8 (calgranulin A)	0.001	2.11
10602372	Alas2	aminolevulinic acid synthase 2, erythroid	0.001	0.69
10375058	Hba-a2	hemoglobin alpha, adult chain 2	0.001	0.20
10444229	H2-DMa	histocompatibility 2, class II, locus DMa	0.001	0.75
10384670			0.001	1.25
10531407	Cxcl9	chemokine (C-X-C motif) ligand 9	0.001	0.56
10341476			0.001	1.48
10580850			0.001	1.38
10375051	Hba-a1	hemoglobin alpha, adult chain 1	0.001	0.17
10563895			0.002	1.26
10582080			0.002	1.22
10435497	Stfa2l1	stefin A2 like 1	0.002	1.97
10420804	Prss55	protease, serine, 55	0.002	0.69
10356510	Iqca	IQ motif containing with AAA domain	0.002	1.42
10338194			0.002	1.72
10515416	Rps8	ribosomal protein S8	0.002	1.34

Affymetrix ID	Gene symbol	Gene name	*P* value	Fold change relative to DOXO
10542164	Clec12a	C-type lectin domain family 12, member a	0.002	0.66
10416923			0.003	1.75
10338145			0.003	0.73
10343850			0.003	0.76
10444291	H2-Ab1	histocompatibility 2, class II antigen A, beta 1	0.003	0.44
10589535	Ngp	neutrophilic granule protein	0.003	2.00
10563597	Saa3	serum amyloid A 3	0.003	1.83
10554032			0.003	1.26
10338338			0.004	1.34
10338345			0.004	0.70
10450154	H2-Aa	histocompatibility 2, class II antigen A, alpha	0.004	0.34
10576437			0.004	1.24
10455866	1700011I03Rik	RIKEN cDNA 1700011I03 gene	0.004	1.53
10343366			0.004	1.49
10343068			0.004	0.71
10379953	4632419I22Rik	RIKEN cDNA 4632419I22 gene	0.004	0.82
10339075			0.004	0.72
10481627	Lcn2	lipocalin 2	0.004	4.92
10513818	Stmn1	stathmin 1	0.004	0.77
10453057	Cyp1b1	cytochrome P450, family 1, subfamily b, polypeptide 1	0.005	1.40
10433885	Cebpd	CCAAT/enhancer binding protein (C/EBP), delta	0.005	1.46
10343183			0.005	0.79
10473444	Aplnr	apelin receptor	0.005	0.58
10342689			0.005	0.70
10343682			0.005	1.31
10343863			0.005	1.42
10458046	D0H4S114	DNA segment, human D4S114	0.005	0.54
10523128	Ppbp	pro-platelet basic protein	0.005	0.72
10502335	Bank1	B-cell scaffold protein with ankyrin repeats 1	0.005	0.77
10342020			0.005	1.78
10341461			0.006	0.71
10555736	Olfr558	olfactory receptor 558	0.006	0.73

Affymetrix ID	Gene symbol	Gene name	*P* value	Fold change relative to DOXO
10453688			0.006	1.73
10398075	Serpina3n	serine (or cysteine) peptidase inhibitor, clade A, member 3N	0.006	4.41
10419934	Myh7	myosin, heavy polypeptide 7, cardiac muscle, beta	0.006	4.56
10342455			0.006	0.74
10401320	Adam4	a disintegrin and metallopeptidase domain 4	0.006	0.76
10492798	Sfrp2	secreted frizzled-related protein 2	0.006	0.83
10404132	Cmah	cytidine monophospho-N-acetylneuraminic acid hydroxylase	0.006	0.82
10434689	Ahsg	alpha-2-HS-glycoprotein	0.006	1.47
10349832	Gm7246	predicted gene 7246	0.006	1.58
10549635	Lilra5	leukocyte immunoglobulin-like receptor, subfamily A (with TM domain), member 5	0.006	0.83
10341250			0.006	0.72
10375608	Scgb3a1	secretoglobin, family 3A, member 1	0.006	1.54
10375559			0.006	1.48
10385507	Gm12185	predicted gene 12185	0.006	0.84
10352125			0.006	0.80
10340250			0.006	1.28
10342135			0.007	1.34
10338699			0.007	1.57
10340559			0.007	1.38
10421970			0.007	0.71
10359888			0.007	1.27
10572083			0.007	1.24
10595145	Gm8074	predicted gene 8074	0.007	1.36
10466130	Ms4a8a	membrane-spanning 4-domains, subfamily A, member 8A	0.007	1.33
10340886			0.008	0.73
10462912	Lgi1	leucine-rich repeat LGI family, member 1	0.008	0.57
10439296	Stfa2	stefin A2	0.008	1.58
10338788			0.008	0.73
10342778			0.008	1.34
10593191			0.008	1.33
10403076			0.008	1.31
10498350	P2ry14	purinergic receptor P2Y, G-protein coupled, 14	0.008	0.77

Affymetrix ID	Gene symbol	Gene name	*P* value	Fold change relative to DOXO
10523359	Cxcl13	chemokine (C-X-C motif) ligand 13	0.008	1.87
10338917			0.008	0.76
10561787	Zfp27	zinc finger protein 27	0.009	0.83
10407387	Gm7120	predicted gene 7120	0.009	0.76
10339214			0.009	1.34
10425283	Maff	v-maf musculoaponeurotic fibrosarcoma oncogene family, protein F (avian)	0.009	1.34
10360418	Rgs7	regulator of G protein signaling 7	0.009	0.84
10342873			0.009	0.66
10368268			0.009	1.21
10439009	Apod	apolipoprotein D	0.009	2.33
10340050			0.009	1.36
10343410			0.009	1.42
10512377	Ccl21a	chemokine (C-C motif) ligand 21A (serine)	0.009	0.62
10408926			0.009	1.20
10338444			0.01	1.59
10338101			0.01	0.76
10342313			0.01	0.75
10595148	Gsta2	glutathione S-transferase, alpha 2 (Yc2)	0.01	1.92
10476321	Prnd	prion protein dublet	0.01	0.68
10499189	Fcrls	Fc receptor-like S, scavenger receptor	0.01	0.77
10448350	Paqr4	progestin and adipoQ receptor family member IV	0.01	0.81
10412481	2410127L17Rik	RIKEN cDNA 2410127L17 gene	0.01	0.78
10483000	Itgb6	integrin beta 6	0.01	0.61
10341946			0.011	1.37
10528909			0.011	1.23
10342440			0.011	1.47
10601581	Trim12c	tripartite motif-containing 12C	0.011	0.80
10342948			0.011	0.74
10476569	2310003L22Rik	RIKEN cDNA 2310003L22 gene	0.011	0.83
10423599	Matn2	matrilin 2	0.011	0.85
10340928			0.011	1.29
10356880	St8sia4	ST8 alpha-N-acetyl-neuraminide alpha-2,8-sialyltransferase 4	0.011	0.76

Affymetrix ID	Gene symbol	Gene name	*P* value	Fold change relative to DOXO
10414175			0.012	1.31
10541614	Clec4d	C-type lectin domain family 4, member d	0.012	1.37
10340650			0.012	0.62
10598976	Timp1	tissue inhibitor of metalloproteinase 1	0.012	1.88
10359908	Rgs4	regulator of G-protein signaling 4	0.012	0.81
10357472	Cxcr4	chemokine (C-X-C motif) receptor 4	0.012	0.84
10417561	Fam107a	family with sequence similarity 107, member A	0.012	1.32
10494388	Hist2h2be	histone cluster 2, H2be	0.012	0.86
10422005			0.012	1.20
10342597			0.012	0.78
10342810			0.012	0.73
10354416			0.012	1.39
10339536			0.012	0.82
10458870			0.012	1.18
10436100	Retnlg	resistin like gamma	0.013	1.64
10339668			0.013	0.79
10339331			0.013	0.77
10443463	Cdkn1a	cyclin-dependent kinase inhibitor 1A (P21)	0.013	2.93
10339921			0.013	0.75
10399751	Colec11	collectin sub-family member 11	0.014	0.84
10441794	Mrgprh	MAS-related GPR, member H	0.014	0.71
10408329			0.014	0.66
10417212	Itgbl1	integrin, beta-like 1	0.014	0.84
10362115	1110021L09Rik	RIKEN cDNA 1110021L09 gene	0.014	0.83
10342360			0.014	0.84
10342631			0.014	0.77

Table S4 Folic acid (FA) effects on gene expression after doxorubicin injection

Affymetrix ID	Gene symbol	Gene name	*P* value	Fold change relative to FA
10341347			0	1.51
10343002			0.001	0.66
10428534	Trps1	trichorhinophalangeal syndrome I (human)	0.002	1.30
10341431			0.002	1.53
10341008			0.003	1.62
10342631			0.003	1.40
10339912			0.004	1.57
10423049	Prlr	prolactin receptor	0.004	1.20
10341724			0.004	1.24
10343682			0.004	0.74
10340000			0.004	1.41
10342884			0.004	1.36
10421970			0.004	1.47
10344580			0.004	1.57
10341795			0.005	1.35
10570735	Defa24	defensin, alpha, 24	0.005	1.26
10409061	Mirlet7f-1	microRNA let7f-1	0.005	1.31
10342619			0.005	1.39
10338869			0.005	1.40
10412481	2410127L17Rik	RIKEN cDNA 2410127L17 gene	0.005	1.34
10343937			0.006	0.73
10338391			0.006	1.38
10414460	Naa30	N(alpha)-acetyltransferase 30, NatC catalytic subunit	0.006	1.24
10339464			0.006	1.40
10339930			0.006	1.51
10339488			0.007	1.35
10343850			0.008	1.28
10371288	Gm1553	predicted gene 1553	0.008	1.36
10340350			0.008	1.59
10338894			0.008	1.49
10339753			0.008	0.67

Affymetrix ID	Gene symbol	Gene name	*P* value	Fold change relative to FA
10503359	C430048L16Rik	RIKEN cDNA C430048L16 gene	0.008	1.28
10344548			0.008	1.40
10338818			0.009	1.45
10343004			0.009	0.74
10340865			0.009	1.28
10484579	Olfr1051	olfactory receptor 1051	0.009	1.44
10339516			0.009	0.76
10473494	Olfr1034	olfactory receptor 1034	0.009	1.60
10343139			0.009	1.41
10338439			0.009	0.75
10404063	Hist1h2ab	histone cluster 1, H2ab	0.009	1.45
10448131	Vmn2r94	vomeronasal 2, receptor 94	0.009	1.35
10342057			0.009	1.40
10341184			0.009	1.38
10339129			0.009	1.44
10401320	Adam4	a disintegrin and metallopeptidase domain 4	0.009	1.31
10344432			0.01	1.44
10339122			0.01	1.44
10565385	Olfr308	olfactory receptor 308	0.01	1.48
10372728	Cdc5l	cell division cycle 5-like (S. pombe)	0.011	1.38
10340698			0.011	1.35
10374842	Ccdc88a	coiled coil domain containing 88A	0.011	1.14
10343058			0.011	1.31
10341149			0.011	1.51
10604053	Nkrf	NF-kappaB repressing factor	0.012	1.17
10338788			0.012	1.38
10503659	Epha7	Eph receptor A7	0.012	1.23
10339429			0.012	1.54
10395612	G2e3	G2/M-phase specific E3 ubiquitin ligase	0.013	1.18
10566686	Olfr490	olfactory receptor 490	0.013	1.35
10435501	Stfa1	stefin A1	0.013	0.74
10344191			0.013	1.42
10343513			0.013	1.40

Affymetrix ID	Gene symbol	Gene name	*P* value	Fold change relative to FA
10341826			0.014	0.77
10341077			0.014	1.34
10473596			0.014	1.40
10342332			0.014	0.73

Table S5 Interaction analysis of sham, doxorubicin (DOXO) and DOXO with folic acid (DOXOFA) on gene expression

Affymetrix ID	Gene symbol	Gene name	*P* value	Log2 mean expression value			Fold change	
				Sham	DOXO	DOXO FA	DOXO vs Sham	DOXO FA vs. DOXO
10566258			0	10.80	6.72	6.81	0.06	1.06
10342109			0.001	6.34	5.51	6.09	0.56	1.49
10598032			0.001	11.29	8.65	8.71	0.16	1.04
10343682			0.002	5.08	5.47	5.04	1.31	0.74
10343850			0.002	5.22	4.82	5.19	0.76	1.29
10341476			0.002	4.24	4.81	4.39	1.49	0.75
10421970			0.002	6.93	6.44	7.00	0.71	1.47
10342631			0.003	5.75	5.37	5.87	0.77	1.41
10341347			0.003	5.76	5.56	6.15	0.87	1.50
10343002			0.003	3.92	4.26	3.67	1.27	0.66
10412481	2410127L17Rik	RIKEN cDNA 2410127L17 gene	0.003	5.80	5.44	5.87	0.78	1.34
10341008			0.003	5.89	5.41	6.10	0.72	1.62
10401320	Adam4	a disintegrin and metallopeptidase domain 4	0.003	6.61	6.22	6.61	0.76	1.31
10574023	Mt2	metallothionein 2	0.003	8.20	11.55	11.03	10.13	0.70
10341431			0.004	6.52	6.17	6.77	0.78	1.52
10338788			0.004	6.45	5.99	6.45	0.73	1.38
10342849			0.004	6.01	6.50	6.36	1.40	0.91
10341795			0.004	6.07	5.76	6.19	0.81	1.35
10342778			0.004	4.21	4.63	4.23	1.34	0.75
10342788			0.005	4.12	4.62	4.42	1.42	0.87
10338145			0.005	6.41	5.95	6.27	0.73	1.25
10339075			0.005	6.73	6.25	6.63	0.72	1.30
10589535	Ngp	neutrophilic granule protein	0.005	5.68	6.68	5.99	2.00	0.62
10341149			0.006	4.81	4.29	4.88	0.70	1.50
10435501	Stfa1	stefin A1	0.006	6.55	6.94	6.52	1.31	0.74
10563597	Saa3	serum amyloid A 3	0.006	6.25	7.13	6.54	1.83	0.66
10379953	4632419I22Rik	RIKEN cDNA 4632419I22 gene	0.006	6.50	6.21	6.42	0.82	1.16
10503359	C430048L16Rik	RIKEN cDNA C430048L16 gene	0.006	6.90	6.62	6.98	0.82	1.29
10342597			0.006	6.11	5.76	6.11	0.79	1.28
10435497	Stfa2l1	stefin A2 like 1	0.006	5.55	6.53	5.99	1.97	0.69
10341184			0.007	6.40	6.03	6.49	0.78	1.38

10338338			0.007	4.68	5.11	4.83	1.34	0.82
10338869			0.007	6.81	6.51	7.00	0.81	1.41
10433885	Cebpd	CCAAT/enhancer binding protein (C/EBP), delta	0.008	6.35	6.90	6.53	1.47	0.77
10555736	Olfr558	olfactory receptor 558	0.008	7.19	6.73	7.06	0.73	1.25
10466130	Ms4a8a	membrane-spanning 4-domains, subfamily A, member 8A	0.008	6.43	6.85	6.53	1.33	0.80
10566254	Hbb-b1	hemoglobin, beta adult major chain	0.009	6.74	5.36	5.37	0.38	1.01
10338444			0.009	4.29	4.96	4.39	1.59	0.68
10338345			0.009	6.51	5.99	6.30	0.70	1.24
10502335	Bank1	B-cell scaffold protein with ankyrin repeats 1	0.009	6.44	6.07	6.32	0.78	1.19
10339122			0.01	5.01	4.64	5.18	0.77	1.45
10341168			0.01	6.72	6.45	6.77	0.83	1.25
10343068			0.01	6.56	6.07	6.37	0.71	1.23
10338629			0.01	6.71	7.03	6.72	1.24	0.81
10399973	Hdac9	histone deacetylase 9	0.01	7.71	7.48	7.74	0.85	1.20
10344143			0.01	6.19	5.81	6.23	0.77	1.34
10399632	F630048H11Rik	RIKEN cDNA F630048H11 gene	0.01	5.81	5.52	5.80	0.82	1.22
10340350			0.01	5.47	5.05	5.72	0.75	1.59
10428534	Trps1	trichorhinophalangeal syndrome I (human)	0.011	6.54	6.41	6.79	0.91	1.30
10513420	Mup7	major urinary protein 7	0.011	4.19	4.64	4.21	1.36	0.74
10340561			0.011	5.01	4.51	4.97	0.71	1.37
10338752			0.011	4.98	4.58	5.05	0.76	1.39
10339168			0.011	6.50	6.18	6.52	0.80	1.26
10417561	Fam107a	family with sequence similarity 107, member A	0.012	6.50	6.90	6.57	1.32	0.80
10604877	Mir465	microRNA 465	0.012	4.29	4.75	4.69	1.37	0.96
10341250			0.012	7.38	6.91	7.21	0.72	1.23
10339921			0.012	6.73	6.32	6.67	0.75	1.27
10375608	Scgb3a1	secretoglobin, family 3A, member 1	0.013	6.63	7.25	6.86	1.53	0.77
10343937			0.013	5.56	5.79	5.33	1.17	0.73
10605820	Zc4h2	zinc finger, C4H2 domain containing	0.013	6.50	6.32	6.52	0.88	1.15
10339488			0.013	5.76	5.53	5.96	0.85	1.35
10439296	Stfa2	stefin A2	0.013	5.35	6.01	5.57	1.58	0.74
10542164	Clec12a	C-type lectin domain family 12, member a	0.013	7.03	6.43	6.68	0.66	1.18
10341724			0.013	6.76	6.63	6.94	0.92	1.24
10342313			0.013	6.65	6.24	6.53	0.75	1.23
10344341			0.014	6.51	6.18	6.58	0.80	1.31
10366737			0.014	7.08	7.33	7.09	1.19	0.85
10375811			0.014	6.61	6.40	6.67	0.86	1.21

10338721<br>10372728<br>10530465	Cdc5l	cell division cycle 5-like (S. pombe)	0.014<br>0.014<br>0.014	4.61<br>8.51<br>4.35	4.21<br>8.22<br>4.70	4.66<br>8.69<br>4.34	0.76<br>0.82<br>1.28	1.36<br>1.38<br>0.78
